# Random Number Generation Based on Heterogeneous Entropy Sources Fusion in Multi-Sensor Networks

**DOI:** 10.3390/s23208497

**Published:** 2023-10-16

**Authors:** Jinxin Zhang, Meng Wu

**Affiliations:** 1 Faculty of Computer and Software Engineering, Huaiyin Institute of Technology, Huaian 223000, China; jarodmars@vip.163.com; 2 School of Computer Science & Technology, Nanjing University of Posts and Telecommunications, Nanjing 210023, China; 3 College of Telecommunications & Information Engineering, Nanjing University of Posts and Telecommunications, Nanjing 210023, China

**Keywords:** multi-sensor networks, information security, entropy, random numbers

## Abstract

The key system serves as a vital foundation for ensuring the security of information systems. In the presence of a large scale of heterogeneous sensors, the use of low-quality keys directly impacts the security of data and user privacy within the sensor network. Therefore, the demand for high-quality keys cannot be underestimated. Random numbers play a fundamental role in the key system, guaranteeing that generated keys possess randomness and unpredictability. To address the issue of random number requirements in multi-sensor network security, this paper introduces a new design approach based on the fusion of chaotic circuits and environmental awareness for the entropy pool. By analyzing potential random source events in the sensor network, a high-quality entropy pool construction is devised. This construction utilizes chaotic circuits and sensor device awareness technology to extract genuinely random events from nature, forming a heterogeneous fusion of a high-quality entropy pool scheme. Comparatively, this proposed scheme outperforms traditional random entropy pool design methods, as it can meet the quantity demands of random entropy sources and significantly enhance the quality of entropy sources, ensuring a robust security foundation for multi-sensor networks.

## 1. Introduction

A multi-sensor network (MSN) is a network system composed of multiple heterogeneous sensors, designed to collaborate in order to collect, process, and transmit various types of information within the environment [1,2]. These sensors encompass diverse types, including image sensors, temperature sensors, sound sensors, motion sensors, and others, capable of gathering a wide range of data such as temperature, humidity, sound, light intensity, and motion patterns [3]. Through collaborative efforts, these sensors can provide a more comprehensive and accurate environmental perception and information acquisition. Coupled with data analysis and processing on relevant platforms, multi-sensor networks have broad applications in fields such as environmental monitoring, smart homes, healthcare, and digital twin [4,5,6,7].

The sensor network, as well as its derivative, the wireless sensor network, along with their integration into the realm of the Internet of Things (IoT), inherently harbor certain intrinsic vulnerabilities. These include susceptibilities such as weak cryptographic passwords, the dearth of periodic patching and updates, insecure application layers, and inadequacies in data protection protocols [8,9]. These vulnerabilities create exploitable opportunities for malicious actors to mount attacks. Contemporary advancements in the field of cryptography offer a robust avenue to fortify the security of sensor networks, with randomness playing a pivotal role as the foundational bedrock of the key management system. To ensure the utmost security of cryptographic frameworks, it is imperative that the random numbers employed possess an exceptional degree of entropy, thus ensuring the preservation of the integrity and uninterrupted availability of the encryption validation ecosystem.

The quality of randomness serves as a pivotal factor determining the quality of an entire key system. As such, a high-quality random source is of utmost significance in the construction of secure and reliable cryptographic keys. The predictability of cryptographic algorithms’ security is significantly compromised when random numbers become foreseeable. In fact, the majority of routines and programming languages generate random numbers through deterministic functions utilizing a random seed, such as an internal clock [10]. Common methods for generating random numbers include [11]: the linear congruential method, the Mersenne Twister algorithm, and the WELL (well-equidistributed long-period linear) algorithm, and the like.

The primary contributions of this paper can be summarized as follows: Firstly, an in-depth analysis of natural sources of randomness, with a particular focus on their application within sensor networks, was conducted. Secondly, an innovative approach was proposed for constructing a high-quality entropy pool by combining chaotic circuits and environmental sensing techniques. This serves to meet the demands for random number generation in the context of multi-sensor network security. Our work is structured as follows: Section “Related Work” discusses the ingestion of randomness in sensor networks. Section 2 is focused on the design of the proposed system and gives details on the entropy pool design. Experimental results are presented in Section 3 and conclusions are in Section 4.

### Related Work

“Randomness” is just a way to refer to systems where the observer cannot predict the outcome with certainty [12]. Computers themselves are incapable of generating truly random numbers; they can only simulate randomness through intricate algorithms. To achieve the generation of high-quality random numbers, it is imperative to acquire a sufficient quantity of randomness from external sources with the requisite quality.

1. Entropy Sources in the Natural World.

In the realm of the natural world, a multitude of stochastic events are inherently present. Through purposeful design, an electronic apparatus can effectively extract these manifestations of randomness and subsequently employ them within domains of heightened security significance, including but not limited to key administration and identity validation. This strategy, involving the harnessing of natural stochasticity to augment security parameters, undeniably crystallized as a pivotal and imperative technological modality within the contemporary landscape of security discourse.

Meteorological data encompass a plethora of factors characterized by stochastic variations, such as temperature, precipitation, wind velocity, and others. It can be conceived as a form of a stochastic entropy source. In practical instances, random.org [13] leverages the randomness generated by atmospheric noise to offer a bona fide random number service.

The intensity and frequency of electromagnetic radiation from celestial bodies such as radiation sources in the universe are subject to stochastic variations, rendering them akin to a stochastic source of randomness. Leveraging the stochastic nature of pulsar pulse flux density, a research team from the Australian National Observatory devised an innovative random number generator [14]. This method successfully underwent validation in accordance with the NSP800 standard [15].

In biological genetics, the combination of genes is subject to stochastic variation, determining the traits and characteristics of organisms. For instance, in the DNA sequence, the arrangement of nucleotide bases denoted as “*s*” can be transformed into a random number through the utilization of a hash function:

(1) $$r=H\left(s\right).$$

Herein, *H* represents a hash function that maps *s* to a random number *r*.

2. Entropy sources within a sensor network ecosystem.

In the context of sensor networks, various sources of randomness also exist. For example:

(1) Genuine randomness of noise: Utilizing a physical circuit with noise to generate random numbers, where noise sources could encompass thermal noise, photoelectric noise, semiconductor device noise, among others. For instance, when powered on, static random access memory (SRAM) populates with random sequences of 0 s and 1 s. This powered-on pattern is unique to each chip, and is thereby feasible for use as a device identifier. This method is referred to as physically unclonable functions (PUF) [16], resulting in high-quality entropy for the generated random numbers.
(2) Inherent randomness of integrated components: Instabilities caused by competition lead to uncertain outputs from logic gates, flip-flops, and triggers. This uncertainty stems from electrical noise within the circuit, rendering the eventual state unpredictable [17].
(3) Genuine randomness of wireless signals: Communication between sensor network devices often employs wireless technology; however, due to environmental influences, wireless signal attenuation and envelope variations are imbued with uncertainty. Hence, in certain wireless communication devices, randomness can be extracted by statistically analyzing received signals, subsequently employed in generating random numbers.
(4) Genuine randomness sensed by environmental sensors: Environmental sensors (such as temperature, humidity, pressure, light, etc.) possess the capability to perceive the ambient environmental conditions. Due to various factors (including weather fluctuations and human interference), these environmental conditions often exhibit inherent randomness. Therefore, by means of analyzing readings from environmental sensors, it becomes possible to extract a measure of randomness, subsequently applied in the generation of random numbers.

3. Entropy source within chaotic circuits.

In electronic devices, randomness can also be extracted through specific circuit designs. Research indicates that chaotic circuits can serve as random number generators due to their inherent uncertainty and sensitivity to initial conditions, thus making them suitable for constructing random number generators based on chaotic systems [18]. Chaotic circuits typically employ nonlinear circuit components such as variable resistors, variable capacitors, operational amplifiers, analog multipliers, etc., and are implemented using analog/digital circuit techniques [19]. This type of random number generator leverages the initial condition sensitivity of chaotic systems and the long-term unpredictability of their behavior, offering distinct advantages over traditional random number generators.

In practical applications, there are two forms of generating random numbers using chaotic systems: one is a true random number generator based on circuit design, and the other is a pseudo-random number generator designed by numerically solving chaotic systems (e.g., Euler’s method, Runge–Kutta method). However, implementing chaotic systems in computers is constrained by computational accuracy, making it challenging to theoretically prove the absence of short-term periodicity in the designed system. To address the precision issues inherent in numerical computation, hardware devices can be employed to implement chaotic systems. This approach helps circumvent the limitations associated with the aforementioned numerical accuracy constraints and potential drawbacks.

## 2. Design of Hybrid Entropy Source Pool

In engineering applications, due to the distinct advantages and limitations of various methods for acquiring randomness, combining them in a cascading manner becomes a practical choice. Thus, a pool of entropy can be established through the fusion of multiple entropy sources. This approach involves collecting input from these sources and injecting the available entropy into the entropy pool to generate non-deterministic random numbers.

The entropy pooling mechanism ensures that the generated random numbers possess a high degree of uncertainty and unpredictability, making it crucial in cryptographic scenarios. These random numbers can be used directly or serve as seeds for cryptographically secure pseudo-random number generators (CSPRNGs). These pseudo-random numbers approach true randomness and exhibit resistance against computational attacks. By utilizing entropy pooling and pseudo-random number algorithms, it becomes possible to obtain high-quality random numbers, thereby providing robust security and cryptographic protection.

**Figure 1:**
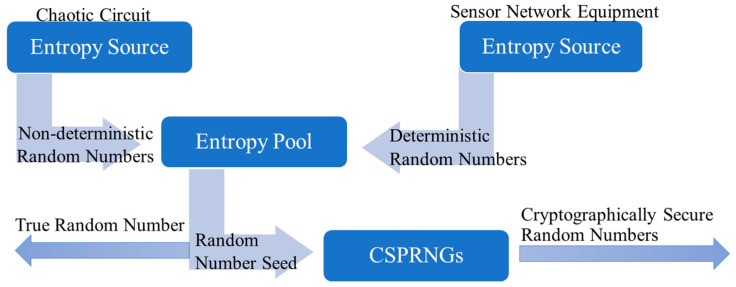
Random number generation model based on entropy pool.

The utilization scenarios of sensor networks are closely aligned with the external natural environment. Consequently, within these scenarios, the perceived information contains a multitude of random factors that can be designed as entropy sources. However, individual random events often fall short of meeting the requirements for both quantity and quality of random numbers. Therefore, this section introduces an entropy source design approach tailored to practical sensor network applications, with the proposed model depicted in Figure 1.

This approach amalgamates the randomness extracted from the environment by sensor network devices with the randomness present in chaotic systems. This fusion overcomes the limitations of inadequate randomness in single entropy source acquisition and the precision deficiencies and short-cycle issues in random number algorithms. This design satisfies the randomness needs of a key system. By employing this hybrid entropy source approach, the high demands for randomness in information systems can be met, yielding a more dependable and secure random number generation.

### 2.1. Chaos Circuit Entropy Source Acquisition

In the chosen design scheme, the Chen chaotic circuit [20] is selected as a category of entropy source. It possesses a more complex topological structure and dynamic behavior compared to the Lorenz chaotic system [21], making it more versatile in domains such as information encryption and secure communication. The description of this chaotic system is as follows:

(2) $$\left\{\begin{array}{c} \dot{x}=a\left(y-x\right),\\ \;\dot{y}=(c-a)x-xz+cy,\\ \dot{z}=xy-bz. \end{array}\right.$$

**Figure 2:**
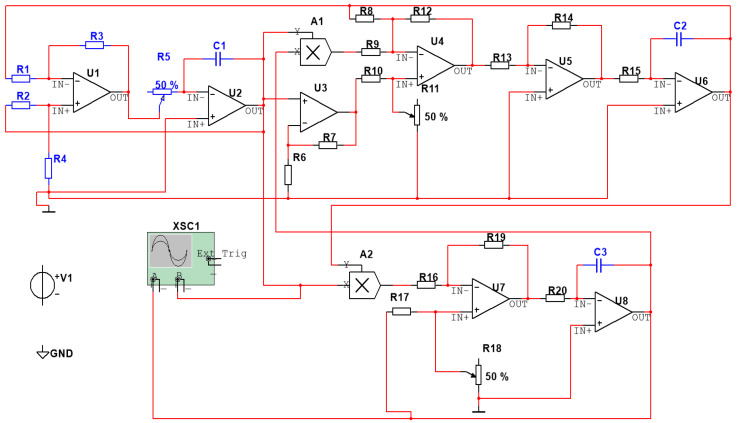
Chen’s chaotic circuit diagram.

**Figure 3:**
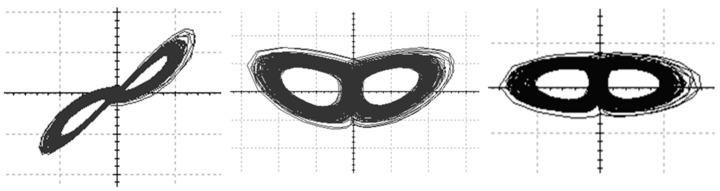
Chen’s chaotic waveform.

The system exhibits intricate nonlinear behavior, including the formation of chaotic attractors and high sensitivity to initial conditions, contingent upon the parameter values such as a,b,and c. Typically implemented as an analog circuit, the Chen chaotic system utilizes operational amplifiers and electronic components to simulate the system’s differential equations. Its application spans diverse fields such as information security, chaotic communication, and random number generation, leveraging its unpredictable and random signal characteristics. The circuit design and parameter selection play a crucial role in shaping the chaotic nature of the system, when a=35,b=3,and c=28, the system exhibits chaotic behavior. The implementation of the Chen chaotic system circuit is depicted in Figure 2, and the phase plot composed of pairs of outputs is illustrated in Figure 3, vividly showcasing the system’s chaotic state. In Figure 3, choose two from the trio of outputs to generate the phase diagrams; these diagrams encapsulate intricate, non-repeating patterns within the system’s dynamic behavior.

Due to the sensitivity of chaotic systems to initial conditions, discrepancies between theoretical designs and practical implementations can arise. Therefore, in the next section, we will delve into the exploration of enriching the entropy pool through the utilization of various types of sensor network application devices.

### 2.2. Entropy Source Acquisition in Sensor Networks

#### 2.2.1. Thermal Noise Amplification Method

The thermal noise amplification method is an effective approach for generating a true random binary sequence by sampling analog signals with quantizing comparators. Thermal noise, also known as white noise, is generated by the thermal agitation of electrons within conductors. It is present in all electronic devices and transmission media. Thermal noise remains unaffected by external biases and is solely determined by the material properties and operating temperature.

The algorithm for random number generation using the thermal noise amplification method is depicted in Algorithm 1. The basic process is as follows:

(a) Generation of thermal noise:

(3) $$V_{n}\left(t\right)=\sqrt{\frac{4k_{B}TR}{\Delta f}}\cdot \eta \left(t\right),$$

where $k_{B}$ represents the Boltzmann constant, T is the absolute temperature, R denotes the input resistance of the amplifier, Δf represents the bandwidth, and η(t) is a Gaussian white noise with zero mean and a variance of 1.

(b) Filtering:
  (4) $$V_{s}\left(t\right)=H\left(f\right)\cdot V_{n}\left(t\right),$$
  where $H\left(f\right)\;$is the frequency response function of the bandpass filter. It refines the signal to a specific frequency range, rendering it suitable for subsequent processing and analysis.
(c) Amplification:
  (5) $$V_{o}\left(t\right)=A\cdot V_{s}\left(t\right),$$
  where A represents the amplification factor.
(d) Sampling and quantization:
  (6) $$x_{i}=Q\left[V_{o}\left(t_{i}\right)\right],$$
  where $t_{i}$ is the sampling moment, and Q is the quantization function.

**Algorithm 1:** Random number generation using thermal noise amplification method1: **Input:** Select the target device as the thermal noise source, set the amplifier gain G, and determine the number of bits N for the quantizer. 2: **Output:** Random number. 3: Collect the voltage or current at the output of the amplifier to obtain the analog noise signal V. 4: $V_{\mathrm{filtered}}$ = Apply filtering process to V; 5: $V_{\mathrm{quantized}}=\mathrm{round}\left(\frac{V_{\mathrm{filtered}}}{\delta }\right);$ //quantization, where δ is the quantization step size 6: $R=\mathrm{binary}\_\mathrm{encode}\left(V_{\mathrm{quantized}},N\right)$; 7: $R_{\mathrm{extracted}}=\mathrm{extract}\_\mathrm{bits}\left(R,M\right)$; 8: **OUTPUT** $R_{\mathrm{extracted}}$.


#### 2.2.2. Video Surveillance Devices

In sensor network devices, a primary functional module involves perceiving the surrounding environment through various types of sensors. Given the prevalence of diverse random factors in the natural environment, it is beneficial to introduce them into the entropy pool to enhance its randomness. This section utilizes video surveillance as an example for the design process.

A camera translates the optical characteristics of a target object into a digital image, and the brightness (pixel value) of each point in the image is incorporated into a sequence, thereby generating a file. If the camera is directed at a dynamic scene, the resulting sequence could exhibit randomness. Similar to the inherent thermal noise in electronic devices, digital images also encompass a noise component. A frame captured by the camera can be expressed as follows:

(7) $$p=p_{NF}+noise.$$

Here, p represents the final image, $p_{NF}$ signifies the noise-free image, and  noise denotes additive noise.

The noise noise can be represented as $\sqrt{It+N_{dt}+N_{t}^{2}}$. This encompasses the following components:

1. Shot noise (photon noise) is described by the quantum nature of light and can be modeled with a Poisson distribution. If the light incident on an optical sensor has a photon flux I, the total number of photons received by the sensor within time t is *It*, and the noise signal is $\sqrt{It}$ [22].
2. Dark noise is generated by electrons within the silicon layer of the sensor itself and is quantified as $N_{d}$, following Poisson statistics. The total noise during time t is $\sqrt{N_{dt}}$.
3. Read–write noise $N_{t}$ arises during data reading and writing between chips and is a transient noise introduced during these processes.

Hence, the signal-to-noise ratio can be expressed as $\frac{It}{\sqrt{It+N_{dt}+N_{t}^{2}}}$ [23].

In recording devices, noise is an inevitable presence. Although these noises might have a detrimental impact on image quality, in the context of this design, they can be harnessed. In actual captured frames, there usually exist changing object components between two frames, and these changes are random in nature. Consequently, these components can be captured and integrated into the design of the entropy pool. By conducting statistical analysis and processing on these changing components, randomness can be extracted and employed for random number generation.

#### 2.2.3. Wireless Intelligent Routing Devices

In sensor networks, extensive employment of wireless communication technologies is observed. Numerous studies harnessed the state of wireless channels to obtain randomness [24,25,26]. These investigations primarily rely on principles such as time variability, channel reciprocity, and spatial decorrelation within the communication process.

By investigating channel characteristics, it is possible to design unpredictable random sources based on correlated features in wireless communication. These sources can be characterized using typical channel parameters. Cooperative relaying and physical layer security networks can utilize wireless broadcast channels, implying that with proper design, cooperative relay network channels can provide a novel and effective approach for extracting randomness.

Intelligent routing devices in sensor networks can effectively amplify and forward wireless signals. The amplify forward (AF) protocol, initially proposed by Laneman [27], is a straightforward relaying cooperation protocol. Relaying techniques are often used in resource-limited or resource-scarce relay nodes. When a relay node receives an attenuated signal from the sender, it amplifies and forwards it to the receiver.

**Figure 4:**
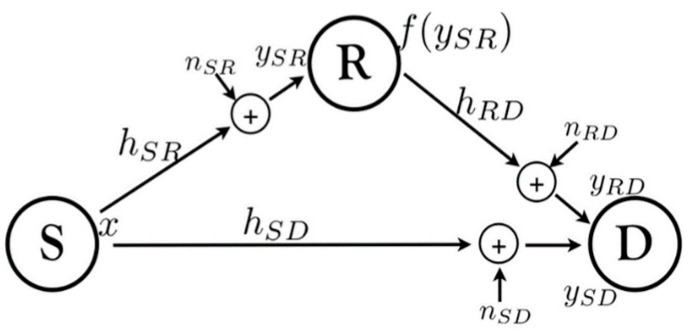
Amplify forward relay model.

Natural noise and interference sources in the environment play a significant role in signal attenuation. In the process of signal amplification and recovery, these random influences are reflected and compensated for. The amplify forward relay model is depicted in Figure 4.

The signals received at the relay node and the receiving node from the source are, respectively, represented as:

(8) $$y_{SR}=\sqrt{P}h_{SR}x+n_{SR},$$

(9) $$y_{SD}=\sqrt{P}h_{SD}x+n_{SD}.$$

In this context, *P* represents the transmitting power of the source; *x* signifies the transmitted signal; and $n_{SR}$ and $n_{SD}$ denote the introduced noise during transmission. $h_{SR}$ and $h_{SD}$ correspond to the channel transfer characteristics between the source and the relay, as well as between the source and the destination node, respectively. The primary function of the amplify and forward (*AF*) relay node involves the amplification of the received signal, which can be mathematically expressed by the following equation:

(10) $$fAF\left(ySR\right)=\beta y_{SR}.$$

β is denoted as an amplification factor subject to an average power constraint on relay transmission. It ensures that the average transmit power at the relay node remains less than or equal to the device power P. As a result, β can be derived using the following formula:

(11) $$E[|f(y_{SR})|2]\leq P,$$

(12) $$E[|\beta y_{SR}|2]\leq P,$$

(13) $$\beta \;\leq \sqrt{\frac{P}{h_{SR}^{2}E\left(x\right)+1}}.$$

When β is set as a constant value, it is referred to as fixed-gain cooperation. On the other hand, when β is a variable, it is termed variable-gain cooperation. By analyzing Equation (9), it can be observed that the value of β is correlated with the channel state $h_{SR}$ from the source to the relay, where channel states possess a stochastic nature. As a result, the value of β can meet the entropy source requirements for key generation. It is worth noting that the amplification factor β is a scalar, which is more readily extractable and exploitable compared to the vector values within the channel transmission characteristics.

In addition to the entropy source mentioned earlier, other sensors within sensor network devices, such as microphones, accelerometers, and gyroscopes, can also contribute valuable randomness. These sensors have the capability to perceive uncertainties and stochastic variations in the environment, which they can then convert into random data. Once various sources of entropy enter the entropy pool, they can be fused using operations such as XOR to enhance the strength and unpredictability of randomness.

### 2.3. The Construction of Entropy Pool

In the system, it is necessary to effectively combine various types of random entropy sources to enhance the randomness and uniformity of collected entropy data and eliminate potential correlations. Common methods include:

Bit operations: The collected entropy data might exhibit patterns or biases. To break these patterns, bit operations are often used to manipulate the data. Bit operations can include bitwise shifts, XOR, AND, OR, and NOT operations. These operations disrupt the bit patterns, increasing randomness.

Hash functions: Hash functions map input data to shorter fixed-length outputs and have the property of uniformly distributing arbitrary-length inputs. In the entropy pool, hash functions can be used to process data, ensuring randomness and uniformity. Common hash functions include MD5, SHA-1, SHA-256, etc.

Confusion algorithms: Confusion algorithms involve complex processing of data to increase randomness and unpredictability. These algorithms can use a series of operations such as computations, permutations, and substitutions to ensure uniform distribution and randomness.

Mixing with historical data: To enhance the complexity of data in the entropy pool, current entropy data can be mixed with previous historical data. This approach can partially eliminate potential temporal correlations and increase the randomness of generated random numbers.

The merits and demerits of the aforementioned methods are delineated in Table 1. These methods offer different trade-offs between simplicity, effectiveness, computational overhead, and impact on system resources. The choice of a mixing method should be based on the specific requirements and constraints of the application at hand.

In the context of sensor networks, when blending the perceived entropy sources, several factors need to be considered. Sensor network devices typically possess limited computational capabilities and storage space. Certain sensor network scenarios might demand real-time generation of random numbers. In such cases, it is essential to choose methods that have low computational overhead and fast generation speed. Given these characteristics, this paper, after comprehensive consideration, selects bit operations as the method for mixing entropy sources to construct the entropy pool.

## 3. System Simulation and Testing

### 3.1. Presentation of Entropy Sources

**Figure 5:**
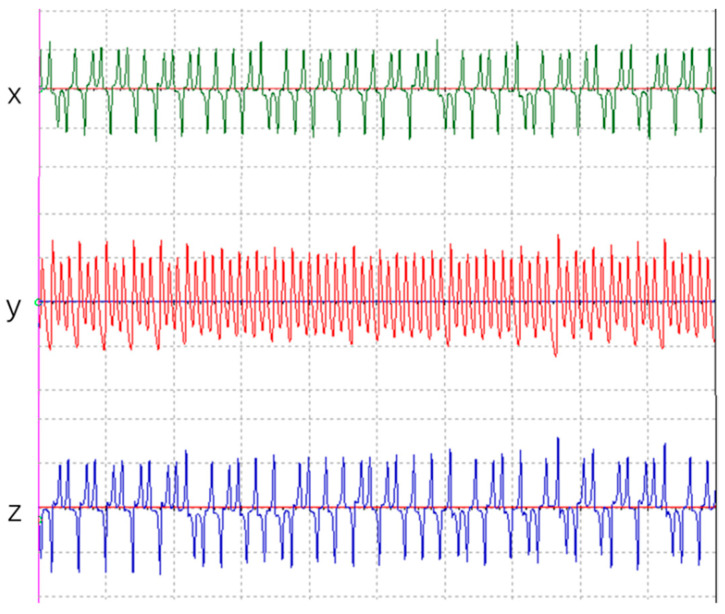
Time-domain plots of chaotic system’s x, y, and z coordinates.

Simulations were conducted on Chen’s chaotic circuit depicted in Figure 2 using Multisim12 software. The component parameters are presented in Table 2. The resulting time-domain plots are illustrated in Figure 5, showcasing distinct temporal characteristics of each output.

To verify thermal noise amplification, this study utilized the/dev/hwrng device within the Raspberry Pi. This device serves as a hardware random number generator, capable of producing high-quality random numbers. The algorithm (Algorithm 2) is as follows:
**Algorithm 2:** Random number generation via thermal noise amplification in Raspberry Pi1: **Input:** num_bytes (number of bytes to read for random number generation). 2: **Output:** output_file (output file path). 3: **dev_random** = OPEN_DEVICE(“/dev/hwrng”); //open the hardware random number generator device. 4: **random_data** = READ_RANDOM_DATA(dev_random, num_bytes); //read random data from the device. 5: **CLOSE_DEVICE** (dev_random); //close the device. 6: **R** = binary_encode (V_quantized, N); //encode the quantized voltage values. 7: **R**_extracted = extract_bits(R, M); //extract the required number of bits as random numbers. 8: **WRITE_TO_FILE** (output_file, random_data); //write the random data to the output file.

**Figure 6:**
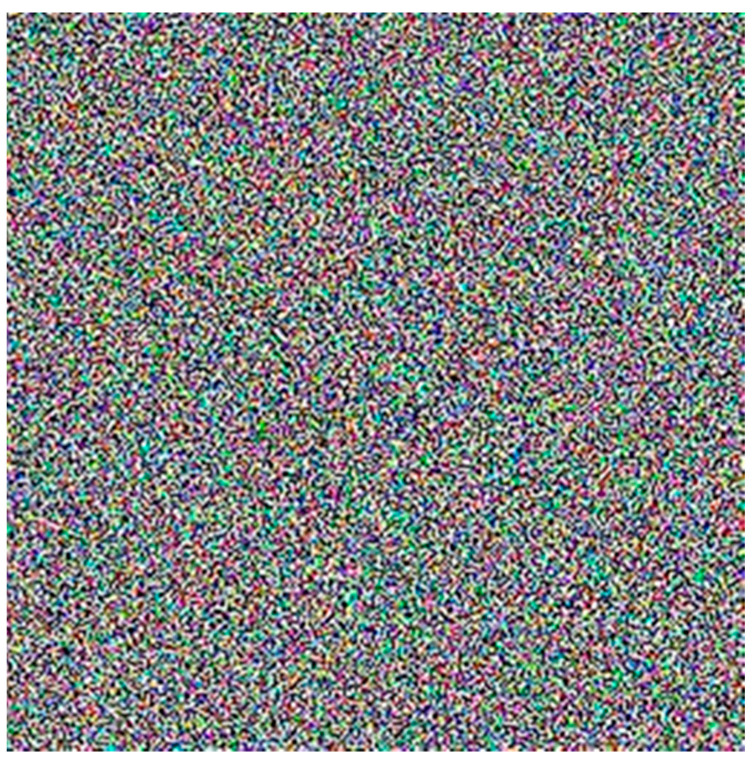
The randomness of thermal noise.

Generate random numbers using Algorithm 2, then create an image matrix of the appropriate size, map binary bits to the 0 to 255 color range based on the number of channels, and represent the data as an RGB image, as illustrated in Figure 6.

**Figure 7:**
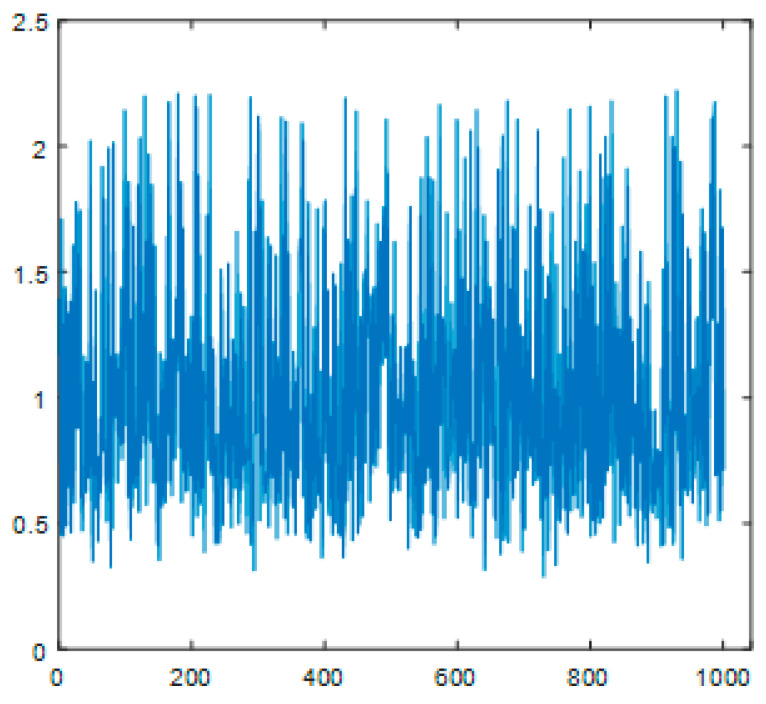
Amplification factor β values at a signal-to-noise ratio (SNR) of 10 dB.

To validate the performance of the proposed intelligent routing randomness extraction method, simulation experiments were conducted using MATLAB regarding the gain parameter β in a wireless channel. It was assumed that both the transmitter and receiver employed digital signal processing, with each number represented using a 10-bit binary format. The channel model used was Gaussian. Using the Monte Carlo method, 1000 experiments (N = 1000) were carried out. The amplification factor β, as illustrated in Figure 7, is characterized by formula (13).

The plots corresponding to Figure 5, Figure 6 and Figure 7 depict the outputs of Chen’s chaotic circuit, thermal noise in electronic terminals, and the forwarding amplification factors in wireless intelligent routing devices. The results suggest that these numerical values manifest a distinctive ‘noise’ characteristic. Upon scrutinizing the images, it becomes apparent that they portray a state of disorder and chaos, making them suitable for key generation purposes.

**Figure 8:**
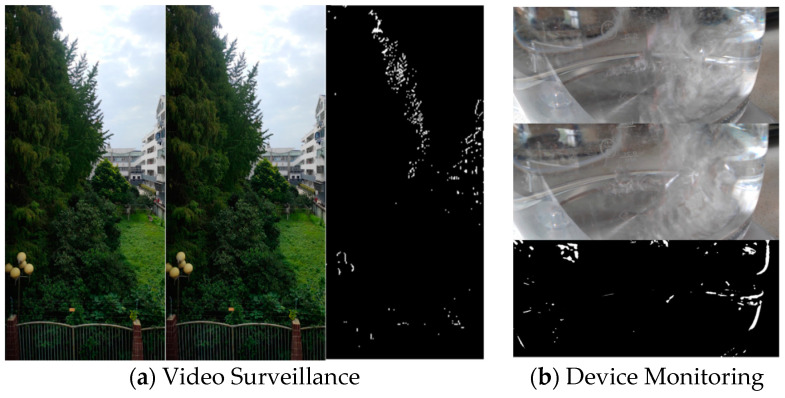
Differences in adjacent 2-s intervals for different monitoring.

The video frames are replete with inherent random variations. Leveraging the OpenCV library, two frames were selected from a surveillance video with a 2-s interval, as shown in Figure 8a. Additionally, observations were made and captured on a heating device, and the changes at 2-s intervals are depicted in Figure 8b.

The video frames under consideration exhibit inherent random variations, presumably stemming from environmental factors such as lighting dynamics, motion, and potential camera perturbations. Employing the OpenCV library, two frames were systematically chosen from a surveillance video, maintaining a temporal interval of 2 s, as elucidated in Figure 8a. Simultaneously, meticulous observations were conducted on a thermal monitoring device, capturing alterations at analogous 2-s intervals, graphically depicted in Figure 8b. A discerning comparative analysis of the two datasets reveals subtle, albeit perceptually imperceptible, stochastic alterations within the same camera’s field of view over a brief temporal span. The proposition emerges to harness these nuanced variations through methodologies such as binary stream conversion. The intended application involves their assimilation as supplementary contributors to an entropy pool, enriching its reservoir of unpredictability for potential cryptographic use cases.

### 3.2. Physical Entropy Source Assessment

For evaluating incoming physical random sources, the average entropy is employed to quantify the average information content of each random datum. In this context, the entropy source S generates random number events represented by the random variable X, with samples denoted as x:

(14) $$H\left(X\right)=-\sum_{x}\mathrm{Pr}\left(X=x\right)\log_{2}\mathrm{Pr}\left(X=x\right).$$

When evaluating the process of random number generation, the adoption of a Markov model is employed. In this context, each newly collected datum is correlated only with the preceding m data points. The concept of average entropy is utilized to express the entropy of each individual datum. Specifically, the average entropy is denoted using the mean symbolic entropy.

(15) $$\bar{H}=\frac{H\left(X_{1},\ldots ,X_{m+1}\right)}{m+1}.$$

The joint entropy of *m* + 1 data points is given by:

(16) $$H\left(X_{1},\ldots ,X_{m}\right)=H\left(X_{1},\ldots ,X_{k}\right)+H\left(X_{k+1},\ldots ,X_{m+1}|X_{1},\ldots ,X_{k}\right)\geq H\left(X_{1},\ldots ,X_{k}\right).$$

The average symbolic entropy can be determined based on the actual data’s compressed bit width *t* and the number of relevant bits *m*. In practical computations, a truncation value *k* (where *k* < *m* + 1) can be designed for assessment. In other words:

(17) $$\bar{H}=\frac{H\left(X_{1},\ldots ,X_{m+1}\right)}{m+1}\approx \frac{H\left(X_{1},\ldots ,X_{k}\right)}{m+1}$$

**Figure 9:**
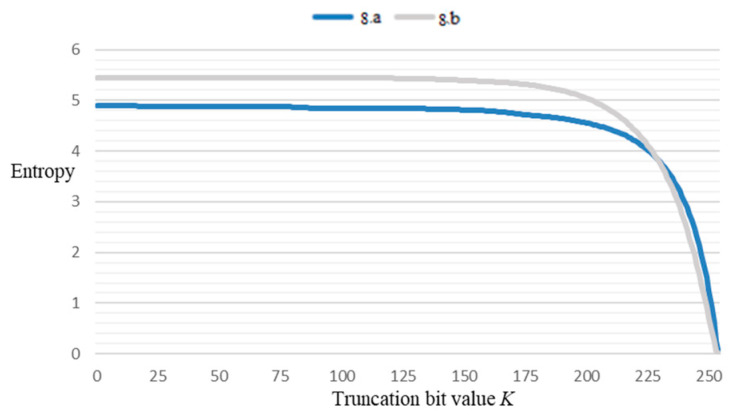
The impact of truncation value *k* on entropy.

Upon performing grayscale transformation on the aforementioned Figure 8, the calculated results are illustrated in Figure 9. It is observable from the outcomes in the figure that as the truncation value k increases, the entropy value of the image exhibits an initial gradual decrease followed by a sharp decline. Consequently, in practical applications, the appropriate truncation bit value can be selected based on the specific circumstances.

### 3.3. Randomness Analysis

Random numbers play a crucial role in the construction of various information systems. When it comes to generating random numbers, chaotic circuits, environmental sensing, and their combination exhibit distinct characteristics. The Table 3 is a detailed comparison.

In order to validate the randomness of generated sequences, it is proposed to combine chaotic circuit signals with physical entropy sources, following the Algorithm 3 below:
**Algorithm 3:** Mixed Entropy Random Number Generation1: **Input:** Chaotic signal, physical entropy sources. 2: **Output:** Random number. 3: **random_seed** = 0; //initialize the random seed. 4: **chaotic_seed** = GENERATE_CHAOTIC_SEED (chaotic_signal); //generate a seed using the chaotic signal as input. 5: **entropy_sources** = GENERATE_ENTROPY_SOURCES(); //generate physical entropy sources. 6: **mixed_seed** = MIX_ENTROPY_SOURCES(chaotic_seed, entropy_sources); //combine multiple entropy sources to create a random seed. 7: **random_number** = GENERATE_RANDOM_NUMBER (mixed_seed); //generate a random number using the mixed seed. 8: **OUTPUT** random_number; //output the random number.

Sensor network systems are composed of a diverse range of sensors and intelligent devices [28]. In this section, we illustrate and compare two application scenarios.

Scenario 1: In the application of sensor network systems, there are numerous video detection devices and wireless transmission switch devices. In this scenario, the amplification factor β from the wireless network and random factors from video images are combined as entropy sources. Random numbers are generated using a random number generation algorithm.

Scenario 2: Considering the demand for random numbers, chaotic circuits are embedded in the devices. These circuits are combined with random factors from video images as entropy sources, and random numbers are generated using a random number generation algorithm.

The scheme employs the random number testing method NIST SP800 [15] provided by the National Institute of Standards and Technology (NIST) for verification. The purpose of this testing methodology is to assess randomness and entropy (i.e., uncertainty of information) and is widely utilized for evaluating and validating the quality of random or pseudorandom number generators. The method encompasses multiple statistical tests covering various statistical features and characteristics, such as uniformity, repetitiveness, and independence between sequences. These tests provide conclusions regarding the sufficiency of randomness or security of the generated sequences.

In statistical testing, the *p*-value is a probability used to determine the likelihood of the hypothesis being true, ranging between 0 and 1. Through testing and comparison, as depicted in Table 4, it is observed that the data generated based on entropy extracted from sensor network devices meet the requirements of randomness. In the proposed chaotic circuit method in this paper, achieving the non-linear parameters in chaos only requires a few operational amplifiers, avoiding the need for more complex multipliers and thus reducing the demand on hardware resources.

## 4. Conclusions

The demand for keys in a sensor network, characterized by a multitude of heterogeneous devices, is both urgent and essential. Various devices require a significant supply of high-quality and efficient keys as their foundation. A high-quality security system not only relies on advanced algorithms, but also relies on high-quality random numbers as its foundation. The natural world is replete with randomness, and sensor networks, with their excellent sensing capabilities, can effectively acquire these sources of entropy, providing valuable ‘raw materials’ for building high-quality security systems based on random numbers. This paper focuses on leveraging the unique characteristics of sensor networks, utilizing simple chaotic circuit designs, and extracting randomness from sensor network sensing devices to create an effective mixed entropy pool. This entropy pool can support high-quality security systems.

## Figures and Tables

**Table 1:** Comparison of various entropy source mixing methods.

Mixing Method	Advantages	Disadvantages
**Bit Operations**	- Simple and fast.	- May not completely eliminate patterns or correlations. - Effect influenced by specific data sets.
**Hash Functions**	- Achieves even distribution via mapping.	- High computational overhead of hash functions. - Potential impact on system performance.
**Confusion Algorithms**	- Provides stronger confusion effect.	- High computational complexity of confusion algorithms. - Demands significant system resources.
**Mixing with Historical Data**	- Eliminates temporal correlations. - Increases randomness and unpredictability.	- Requires maintenance and management of historical data. - Potential high storage consumption.

**Table 2:** Component list.

Description	Quantity	Identifier
OPAMP_3T_VIRTUAL	8	U1, U2, U3, U4, U5, U6, U7, U8
MULTIPLIER, 1 *v*/*v* 0 V	2	A1, A2
RESISTOR, 100 kΩ 5%	7	R6, R7, R13, R14, R16, R17, R19
RESISTOR, 200 kΩ 5%	1	R8
RESISTOR, 10 kΩ 5%	2	R9, R12
RESISTOR, 49.9 kΩ 5%	1	R10
POTENTIOMETER, 100 kΩ	1	R11
RESISTOR, 40.2 kΩ 5%	1	R15
POTENTIOMETER, 66.5 kΩ	1	R18
RESISTOR, 158 kΩ 5%	1	R20
DC_POWER, 5 V	1	V1
POWER_SOURCES, DGND	1	GND

**Table 3:** Comprehensive comparison of random number generation approaches.

Features	Chaotic Circuits	Environmental Sensing	Combined Approach
Nature of Randomness	Generates pseudo-random numbers based on complex nonlinear dynamics.	Produces true random numbers based on natural environmental variations.	Integrates pseudo-random and true random, allowing adjustable balance.
Controllability	Controllable through parameter adjustments.	Dependent on natural environmental changes.	Adjustable mix to control the balance.
Stability	Sensitive to initial conditions but stable outputs with parameter tuning.	Susceptible to environmental fluctuations, may result in unstable outputs.	Balances the instability of chaos with uncertainty of environmental sensing.
Quantity	Large quantity, capable of producing a significant number of pseudo-random numbers.	Limited by environmental variations, may result in a restricted quantity.	Adjustable mix to balance a large quantity of pseudo-random with a relatively smaller quantity of true random numbers.
Complexity	Built upon complex mathematical models.	Relies on natural environmental changes, avoiding complex algorithms.	Requires consideration of the complexities of chaotic circuits and environmental sensing.
Applicability	Suitable for scenarios requiring controllable, large quantities of pseudo-randomness.	Suitable for scenarios demanding genuine, high-quality randomness.	Adaptable to diverse scenarios by choosing an optimal random number generation strategy based on specific needs.

**Table 4:** *p*-value comparison.

Statistical Test	Scenario 1 *p*-Value	Scenario 2 *p*-Value	Huang et al. [29] *p*-Value	Tuncer et al. [30] *p*-Value	Results
Frequency Test	0.226567654	0.616726644	0.322332	0.757	Pass
Block Frequency Test	0.05437972	0.758595049	0.155513	0.135	Pass
Run Test	0.048433065	0.0421974	0.979957	0.801	Pass
Longest Run in a Block Test	0.609463496	0.421431103	0.951515	0.497	Pass
Binary Matrix Rank Test	0.924208024	0.181719167	0.767895	0.336	Pass
Discrete Fourier Transform Test	0.347599557	0.542408684	0.42673	0.501	Pass
Non-overlapping Template Matching Test	0.993558299	0.999687692	0.999103	0.698	Pass
Overlapping Template Matching Test	0.944470195	0.987985525	0.640417	0.63	Pass
Maurer’s Universal Statistical Test	0.329927487	0.310222462	0.999914	0.435	Pass
Linear Complexity Test	0.759038639	0.722101991	0.351439	0.644	Pass
Serial Test	0.103259524	0.622267693	0.18509	0.444	Pass
Approximate Entropy Test	0.10352127	0.622150981	0.319519	0.949	Pass
Cumulative Sums Test	0.314853196	0.319493648	0.312105	0.88	Pass
Random Excursions Test	0.047498605	0.117704333	0.072597	0.77	Pass
Random Excursions Variant Test	0.113909966	0.207932487	0.480935	0.478	Pass

## Data Availability

No new data were created.
